# Follicular Fluid redox involvement for ovarian follicle growth

**DOI:** 10.1186/s13048-017-0342-3

**Published:** 2017-07-12

**Authors:** Cláudia Freitas, Ana Catarina Neto, Liliana Matos, Elisabete Silva, Ângela Ribeiro, João Luís Silva-Carvalho, Henrique Almeida

**Affiliations:** 1 Reproductive Medicine, Obstetrics and Gynaecology, Hospital Dr. Nélio Mendonça, SESARAM, Funchal, Portugal; 2 Departamento de Ginecologia e Obstetrícia, Faculdade de Medicina, Universidade do Porto, Porto, Portugal; 3 IBMC - Instituto de Biologia Molecular e Celular and Instituto de Investigação e Inovação em Saúde - i3S, Universidade do Porto, Porto, Portugal; 4 Ageing and Stress Group, Experimental Biology Unit - Department of Biomedicine, Faculdade de Medicina, Universidade do Porto, Porto, Portugal; 5 Faculdade de Ciências da Nutrição e Alimentação and Faculdade de Medicina Dentária, Universidade do Porto, Porto, Portugal; 6 CETI – Centro de Estudo e Tratamento da Infertilidade, Porto, Portugal; 7 Obstetrics and Gynaecology, Hospital CUF-Porto, 4100 180 Porto, Portugal

**Keywords:** *Cumulus Oophorus* cells, Follicular fluid, Oocyte maturation, Oxidative stress

## Abstract

As the human ovarian follicle enlarges in the course of a regular cycle or following controlled ovarian stimulation, the changes in its structure reveal the oocyte environment composed of cumulus oophorus cells and the follicular fluid (FF).

In contrast to the dynamic nature of cells, the fluid compartment appears as a reservoir rich in biomolecules. In some aspects, it is similar to the plasma, but it also exhibits differences that likely relate to its specific localization around the oocyte. The chemical composition indicates that the follicular fluid is able to detect and buffer excessive amounts of reactive oxygen species, employing a variety of antioxidants, some of them components of the intracellular milieu.

An important part is played by albumin through specific cysteine residues. But the fluid contains other molecules whose cysteine residues may be involved in sensing and buffering the local oxidative conditions. How these molecules are recruited and regulated to intervene such process is unknown but it is a critical issue in reproduction.

In fact, important proteins in the FF, that regulate follicle growth and oocyte quality, exhibit cysteine residues at specific points, whose untoward oxidation would result in functional loss. Therefore, preservation of controlled oxidative conditions in the FF is a requirement for the fine-tuned oocyte maturation process. In contrast, its disturbance enhances the susceptibility to the establishment of reproductive disorders that would require the intervention of reproductive medicine technology.

## Background

The ovarian follicle is the center of a complex network of systemic and local signaling pathways [1–3] whereby the follicle grows and the oocyte acquires maturity, which is necessary for fertilization to occur and for a viable embryo to develop.

**Fig. 1:**
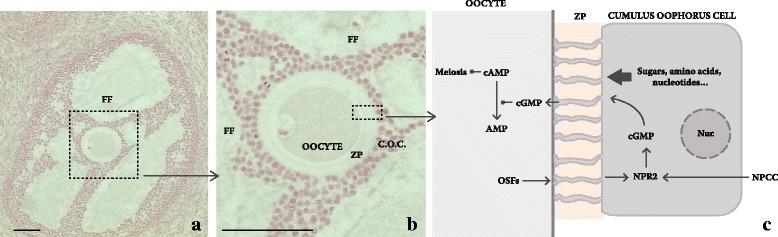
Essential structure of the follicle and simplified overview of cumulus oophorus cell and oocyte interaction. **a** Advanced pre-antral stage follicle, showing the two compartments: the antrum containing the follicular fluid (FF) and the cumulus oophorus (c.o.) cells around the oocyte. **b** The magnification shows the relation of zona pellucida (z.p.) to the oocyte and the c.o. cells. *C. granulosa* cell natriuretic peptide type C (NAPC) interaction with the c.o. cells NPR2 receptor that is promoted by oocyte secretory factors (OSFs) results in cGMP enhanced synthesis and its transport to oocytes along the extensions of c. o. cells that protrude through the zona pellucida (ZP). In the oocyte, it blocks phosphodiesterase-3A, which maintains cAMP high and the oocyte in prophase I arrest. Rabbit ovary. Bar in A and B: 100 μm

This is a long process that in its early course does not exhibit striking structural features, apart from enlargement of oocyte volume and an increase in the number of its surrounding cells. Then, a distinct cellular and liquid environment is established and encircles the evolving oocyte. It consists mainly of *cumulus oophorus* (c.o.) cells and the follicular fluid (FF), that occupies the antrum (Fig. 1a, b).

The complexity of the structural and functional changes observed in this tissue, that comprises cells with different lineages, and the remarkable mixture of biomolecules present in the FF is by itself surprising. But, how the regulation is carried out in order to result in ovulation of a good quality oocyte, is extraordinary. The purpose of this review is to focus on a part of this regulation.

### The follicular microenvironment – *Cumulus oophorus* cells

C.o. cells are a structural feature of the antral follicle, easily identifiable because they immediately envelope the oocyte. Despite the difference in cell lineage, their anatomical proximity and the mutual, paracrine actions they perform [4, 5] have contributed to the establishment of the concept of a single entity, designated cumulus oocyte complex (COC).

The c.o. cells originate from the structurally homogeneous granulosa cell layers of the pre-antral follicles; however, during follicle growth, they become functionally distinct from the mural granulosa cell counterparts as they exhibit different proliferative and steroid secretory abilities, in an oocyte dependent manner [6]. In addition, c.o. cells have cytoplasmic extensions, whose tips, upon protruding through the zona pellucida, make direct contact with the oocyte cell membrane by way of gap junctions [4]. In their molecular organization, small calibre molecular channels connect both cytoplasms across the intercellular space; however, while the channels limit the passage of large molecules, they allow the small ones to flow and exert actions in the target cell. As consequence, c.o. cells become the source of small nutrients to the oocyte, as sugars, amino acids and nucleotides [7], and they are also the origin of signal molecules that regulate the progression of meiosis. Moreover, c.o. cells supply the oocyte with reduced glutathione, which is a major intracellular antioxidant [8, 9], and with cysteine for additional glutathione synthesis [10]. C.o. cells thus afford protection to the oocyte, notably from oxidative stress [11, 12].

The communication c.o. cells/oocyte is not a one-way communication. Indeed, the structural organization of the COC allows transportation of molecules in both directions, implying that signals originated in the oocyte may interact with appropriate receptors on c.o. cells and modulate their function. In fact, it was reported that while removal of the oocyte from the COCs holds proliferative and secretory abilities of c.o. cells, resuming the contact with the oocyte resulted in the regaining of those properties [6]. These and other previous findings favoured the existence of diffusible oocyte secretory factors (OSFs) that act on the c.o. cells and modulate their proliferative and secretory abilities [13, 14]. OSFs behave as growth factors and include the growth differentiation factor 9, GDF9, and the structurally related bone morphogenetic protein 15 (BMP15, former GDF-9B), members of the Transforming Growth Factor Beta (TGFβ), superfamily [15]. Usually, this group of proteins exhibits a set of seven conserved cysteine residues at the mature region but both GDF-9 and BMP-15 have only six residues of the seven-cysteine set; moreover, BMP15 contains two additional cysteine residues upstream of the first conserved cysteine [16]. While, the absence of one cysteine in GDF9 and BMP15 restrains their ability to establish covalent binding and dimerize, their non-covalent binding still results in functional homo or heterodimers [15]. Interestingly, the heterodimer arrangement shows stronger biological activity compared to the homodimers and appears to promote a different Type 1 and Type 2 TGFβ receptor assembly [17, 18].

The importance of these factors for normal follicle development is high. The ovaries of *Gdf9* knockout mice have normal structure but the follicles do not develop beyond the primary follicle stage [19] and granulosa cells proliferative response is weaker [20]. *Bmp15* knockout female mice have ovulation disturbances and smaller COCs [21].

Upon OSFs interaction with c.o. cell membrane receptors, a transductive pathway is initiated, involving SMAD factors [15, 22]. Following their translocation to the nucleus, they function as transcription factors and activate genes involved in c.o. cell proliferation [13] and glycolysis for pyruvate production [23], extracellular matrix expansion [24], cholesterol synthesis [25] and intra-oocyte glutathione level regulation [26].

OSFs are thought to be determinant for the establishment of c.o. cells lineage, with implications to c.o. cells proliferation and metabolism [27]. However, recent evidence favors the existence of other determinants of c.o. cells distinct features as cell response may differ according to the size of the follicle [28].

An important effect of OSFs is to promote c.o. cells NPR2 receptor expression which, when activated by the granulosa cell Natriuretic peptide type C, enhances c.o. cells cyclic Guanosine Monophosphate (cGMP) synthesis; in turn, cGMP is transported across the gap junctions to the oocyte where it inhibits phosphodiesterase 3A and thus prevents cyclic Adenosine Monophosphate (cAMP) hydrolysis. While cAMP level is high, the oocyte is arrested in prophase I [29]; however, when LH surge ensues, cGMP flow to the oocyte is blocked, which results in intra-oocyte drop of cAMP level and release of the break on meiosis (Fig. 1c).

These fine-tuned c.o. cells actions, whose end-point is successful fertilization, suggest that a change in their structural arrangement around the oocyte or in their function might be related to infertility. Indeed, the description of c.o. cells structural properties and the assignment of a quantitative score thereof, have been used to predict fertilization ability [30] although the subjective nature of the interpretation has posed natural accuracy concerns [31].

### The follicular microenvironment – Follicular Fluid

The presence of FF, the other component of the oocyte microenvironment, is noticed early in the pre-antral stage of the ovarian follicle when small, apparently empty spaces appear between granulosa cells (Fig. 1a, b). During the following months, as the follicles grow, to reach 20 mm of diameter and more in the case the dominant follicle stage is attained [1], the FF occupies a single compartment that enlarges progressively until it surrounds most of the c.o. cells and the oocyte.

The FF contains a complex mixture of steroids, metabolites, polysaccharides, proteins and small peptides, reactive oxygen species (ROS) and antioxidant enzymes [32, 33]. Probably, apart from acting directly as cellular signals, the mutual interaction of many of these FF molecules, or their absence, will also contribute to appropriate follicular growth and oocyte maturation.

The identification of FF peptides and related proteins has been the subject of intense research. In the last two decades, the emergence of powerful techniques for protein analysis, as 2D PAGE combined with MALDI-MS or liquid chromatography MS/MS, opened wide perspectives for the study of the oocyte liquid environment by identifying a large number of FF proteins.

Nearly 50% of FF proteins have extracellular origin; most of the remaining proteins can be related to organelles or other cell features; regarding their biological activities, about 20% are enzymes, 14% are part of the extracellular matrix or are involved in cell adhesion, and another 16% include regulatory proteins as growth factors, protease inhibitors, transcription factors and other signaling related molecules [32]. Therefore, the FF compartment has a remarkable diversity of biomolecules; however, the fact that many of the proteins are enzymes, suggests that the compartment is not a mere reservoir but also supports intense metabolic activity, with impact on c.o. cells functioning and oocyte fate.

Angelucci et al. reported 695 different FF proteins or peptides, of which 625 were common to plasma and 27 FF additional proteins were considered absent from the plasma [34]; these figures were enlarged to over 3700 peptides but only 480 proteins received firm identification [32]. When these findings were combined with previous data [34, 35] an impressive list of 789 different FF proteins was generated that included 64 FF specific proteins [32].

Many of the identified proteins relate to biological processes as acute response signaling, inflammation, coagulation and complement cascades. In this respect, plasma and FF seem indistinguishable [34–36], indicating that although they are localized in distinct compartments, interchange between them exists. This is not unexpected because there is a rich vascular network around the growing follicle [37] that favors plasma proteins movement across the vessel walls into the FF compartment [38]. Whether some selectivity exists is controversial; while some suggest it [38], others argue that there is no control on the passage of molecules when ovulation approaches [39], supporting the view that the process has similarities with an inflammatory reaction [40].

The blood compartment is not the sole source of biomolecules to the FF because proteins also originate in mural granulosa cells, the c.o. cells and the oocyte [7, 32]. Assuming that identification of new FF molecules will continue, it is likely that other, low weight or low concentrated peptides will be identified in the future and, eventually, be ascribed to reproductive disorders.

The delicate nature of molecular interactions that fine tune the COC functioning, as long as the follicle grows until ovulation, requires an appropriate environment that is thus provided by the local organization and interaction of c.o. cells and the FF. However, these players have a profound difference: while cells are efficient and adaptable entities, albeit small, the FF compartment is a reservoir with limited versatility. So, to maintain a balance in a complex mixture of biomolecules, it is reasonable to admit that important local chemical buffering systems are required.

Redox reactions and their regulators are likely to fit into this requirement. On the one hand, redox reactions are at the foundation of cell metabolism and, therefore, are a basic condition for life existence; in addition, their regulated intervention is important for a variety of reproductive processes and their dysregulation seems to contribute to reproductive disorders [41]. Furthermore, important players involved in redox balance were identified in FF composition [34].

### Oxidation and effects of oxidants on biomolecules. Antioxidant balance

**Fig. 2:**
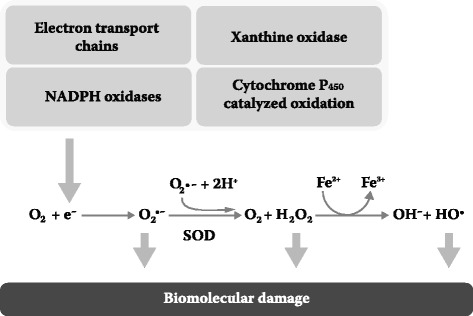
Endogenous production of superoxide. Electrons generated in mitochondria, endoplasmic reticulum and cell membrane electron transport chains, and oxidation catalyzed by a diversity of enzymes, partially reduce oxygen into superoxide anion (O_2_^•^–,); this is dismutated by superoxide dismutase (SOD) into O_2_ and H_2_O_2_; metal mediated catalysis of H_2_O_2_ originates the strongly reactive hydroxyl radical (HO^•^). When targeting biomolecules, the three oxidants, as well as many other resulting from the catalytic action of xanthine oxidase, cyclooxygenases and lipoxygenases, nitric oxide synthase, norepinephrine breakdown and autoxidation processes, enlarge the diversity of oxidants and cause biomolecule damage (reviewed in ref. [43])

Among the oxidants that affect cell functioning, ROS are compounds inevitably produced in the course of cell metabolism. The primary oxidant is superoxide, O_2_^•^- (Fig. 2), that is rapidly dismutated by superoxide dismutase (SOD) into hydrogen peroxide (H_2_O_2_). Either by itself or upon conversion into more reactive species, their continued production imposes an oxidative burden on cells and harmful consequences in biomolecules as sugars, nucleic acids, lipids and proteins.

Briefly, polysaccharide oxidation results in polymerisation loss [42], the formation of advanced-glycation end products (AGEs) when reacting with amino groups, and further oxidation when AGEs interact with their receptors [43]. In nucleic acids, ROS target their sugar moieties, purines and pyrimidines, resulting in strand breaks, base deamination or loss, and adduct formation. Together with the failure of repair mechanisms, DNA may be irreversibly damaged due to the establishment of mutations and result in cell death. In lipids, the C = C double bonds of unsaturated fatty acids are important ROS targets; by continuously forming peroxyl radicals, they extend the damage to nearby lipids, produce more hydroperoxides and affect membrane structure and functional properties.

Proteins are another category of biomolecules subjected to oxidation. The effect of ROS on them is diverse, and relates to the intensity of the challenge, the oxidized amino acids and the type of protein involved [44]. Cysteine and methionine are very sensitive to oxidation, but are amenable to revert on account of reductive enzymes [45]. A particularly important effect is carbonylation which causes irreversible damage by exposing hydrophobic residues previously buried in the protein structure. All these changes lead to the unwanted state of oxidative stress, in which there are too many ROS in relation to the available antioxidants [43].

Fortunately, organisms are endowed with intracellular and extracellular antioxidants whose effect is to delay, prevent or remove oxidative damage to a target molecule [46]. They may be enzymes, the most important of which are SOD isoforms, catalase (CAT) and glutathione peroxidase (GPx). The metalloenzyme SOD promotes the dismutation of O_2_^•^- to O_2_ and H_2_O_2_ and is considered the first defense line against ROS by way of three mammalian isoforms: the cytosolic copper zinc SOD (CuZnSOD or SOD1) [47], the mitochondrial manganese SOD (MnSOD or SOD2) [48] and the extracellular copper and zinc containing SOD (EC-SOD or SOD3) [49]. The action of SOD enzymes results in enhanced H_2_O_2_ production, controllable by CAT and GPx activity [50]. CAT localizes mainly to peroxisomes, where they convert H_2_O_2_ to water and molecular oxygen [51]. In turn, there are four human GPx isoenzymes: the GPx1, cytosolic and mitochondrial, the cytosolic GPx2, the extracellular GPx3 and the phospholipid hydroperoxide GPx4 [52]. They neutralize H_2_O_2_ or organic peroxides to water or alcohol, while converting reduced glutathione (GSH) to oxidized glutathione (GSSG) [53].

In addition, other non-enzymatic antioxidant compounds are endogenously synthesized or acquired from the diet. Coenzyme Q10 (CoQ), uric acid (UA) and GSH are examples of endogenous antioxidants. CoQ, which also plays a central role in the mitochondrial respiratory chain, is an antioxidant that prevents or neutralizes lipid peroxyl radicals and regenerates vitamin E [54]. In humans, UA acts as a strong scavenger of singlet oxygen and hydroxyl radicals and is oxidized mainly to allantoin [55]. Apart from its participation in enzymatic reactions, GSH is a direct, potent scavenger of free radicals; it is oxidized to GSSG and is subsequently reduced to GSH by glutathione reductase, thus maintaining cellular supply of GSH [56]. Nutrient antioxidants include ascorbic acid (vitamin C), naturally present in fresh vegetables and in some mammals. Primates lack the enzyme that catalyzes the last step in ascorbate synthesis [57]. The biologically active form, L-ascorbic acid, is a strong reducing agent that readily neutralizes free radicals and harmful ROS, such as the hydroxyl radical, H_2_O_2_ and singlet oxygen [58]. The fat-soluble alpha-tocopherol (vitamin E) has high antioxidant potency, particularly as cell membrane protector. It prevents lipid peroxidation by scavenging peroxyl, oxygen and superoxide anion radicals while converting itself to the alpha-tocopherol radical that may be reduced back to its original form by ascorbic acid, CoQ or other cellular reducing compounds [59]. Folate is another important free radical scavenger with antioxidant properties [60]. UA, ascorbate and tocopherol plasma levels are reflected in the frequently used assay of the total antioxidant capacity, TAC [61].

### Follicular Fluid redox activity involvement in reproductive modulation

ROS are usually considered harmful agents because their effects modify the structure and functioning of biomolecules but some ROS are also useful signalling agents.

Hydrogen peroxide is at the centre of such processes. Following early reports showing that H_2_O_2_ is required for growth factor signal transduction [62], the scope of ROS involvement in signalling increased. The multifaceted reproductive system is an example of the expansion of ROS physiological and regulatory roles to folliculogenesis, oocyte maturation, luteal regression and fertilization [41]. In fact, H_2_O_2_ appears to intermediate crucial changes as is c. o. enlargement, that occurs during the pre-ovulatory LH surge, which can be mimicked by the addition of H_2_O_2_ and is amenable to inhibition by antioxidants [63]. Less clear is the limit between signalling and unwanted or harmful consequences. In a selected process as oocyte maturation, it was estimated that 60 ng of ROS/oocyte maintains it in diplotene arrest, whereas a moderate increase in ROS production to 80 ng/oocyte is able to resume meiosis [64].

It has been recognized that important targets of signalling ROS are thiol groups of «free» cysteine residues, not involved in the establishment of disulphide bonds [65]; these are widespread at the surface of proteins and may be oxidized rapidly, but reversibly, thus contributing to attenuate the oxidative insult and to prevent its spread along the entire protein [65]. The diversity of modifications exhibited by oxidized cysteine thiol group [66] is evidence of its versatility and ability to acquire unique functional properties [67]. Such modifications, apart from the simple disulfide bond formation, include the glutathionylation and nitrosylation and other forms of increasing thiol level of oxidation as are the sulfenic, the sulfinic and the sulfonic acid modifications [66, 67].

Not all cysteine residues are regulatory targets [65, 68]. However, there is evidence that different categories of proteins are susceptible to oxidation through cysteine thiol groups and it is likely that different experimental conditions will produce specific sets of intracellular or secreted modified proteins [66, 67]. Susceptible proteins include tyrosine phosphatases, proteases as caspases, adaptors and chaperones as heat shock proteins, transcription factors as the bacterial OxyR protein, p53, hypoxia-inducible factor, redox modulator proteins, as thioredoxin and peroxiredoxin, and other intracellular and extracellular proteins as albumin, transthyretin, high-mobility group protein box 1 (HMGB1), profilin and vimentin [66, 67]. As even non-active residues, may also be targeted by oxidation, provided they are localized in a susceptible position, it was estimated that >500 proteins are in such condition [69], thus making the oxidation of cysteine thiols a process with the potential to affect a variety of biological functions.

In contrast to the intracellular compartment, extracellular spaces such as plasma and FF compartments are oxidizing environments that affect their contents and, in particular, contribute to the diminished ratio of reduced cysteine. The condition results in near absence of free cysteine [66] and in the decrement of free thiols of the FF compartment, which appears critical for the redox balance and has strong implication to the reproductive outcome [70].

The exposure to an oxidative environment of the OSFs BMP15 and GDF9 present in the FF suggests that the thiols from cysteine residues at the pro-region are susceptible to oxidize. Although this event may prevent the oxidation of the remaining molecule, it would do so at the expense of the OSFs ability to dimerize or heterodimerize [17] and interact with their c.o. cells receptors, which is a necessary step to promote COC development and oocyte maturity.

FF samples obtained from women upon controlled ovarian stimulation contain common antioxidants as SOD isoforms 1, 2 and 3, GPx, CAT, glutathione S-transferase (GST), peroxiredoxins (Prx) and glutathione reductase [32, 34, 35]. Beyond their presence, enzyme activity was also detected [71, 72], favouring the existence of an ongoing antioxidation in the FF, eventually intensified close to ovulation when follicles enlarge considerably [73]. Other, important antioxidants as GSH, vitamin E and SOD were assayed in the FF and their levels were found to parallel serum levels [74]. These findings further support the view that an effective communication between FF and plasma compartments exists and that understanding the redox regulatory mechanisms in plasma will improve the knowledge on similar mechanisms operating in FF.

Albumin, a major component of both the serum and the FF, is well known for its role in osmotic regulation. In addition, albumin has antioxidant properties. In fact, its structure includes a cysteine in position 34, not engaged in disulfide bonding, whose thiol group is able to react reversibly with oxidizing species; moreover, because of its abundance, albumin behaves as antioxidant and oxidation sensor [65]. Interestingly, although most serum and FF albumin is detected in its reduced form, the relative concentration of reduced albumin is higher in FF compared to the serum [75] suggesting that FF albumin stands as an important means to buffer oxidative conditions.

The level of reduced albumin in FF has positive reproductive impact as antioxidant. In fact, FF samples associated to viable oocytes, exhibited significantly higher level of reduced albumin, compared to FF samples surrounding structurally degenerated oocytes, whose level of oxidized albumin was higher [75]. Later in development, oxidation in the FF maintains its relevance; it was reported that oocytes resulting in low quality embryos, had been retrieved from FF containing higher H_2_O_2_ level, compared to oocytes that resulted in high quality embryos [76]. Moreover, oxidized methionine and ubiquitinated proteins were reported to be increased in FF from women whose pregnancy was unsuccessful [77], further emphasizing the irreversibility of the damaging effects of continued oxidation [78] and the functional disability therein. Therefore, enhanced oxidation in the FF results in lesser reproductive success and, in fact, lower level of ROS and higher total antioxidant capacity (TAC) were recognized as pregnancy predictors for intracytoplasmic sperm injection [79].

Although much of the stress is attributed to increased ROS production [80, 81], it is also consequent to local decrement of antioxidant level. Higher FF concentrations of vitamin E and beta carotene (and ascorbic acid to a lesser extent) were associated with better pregnancy outcome [82], whereas the level of antioxidants was smaller in unexplained infertility and other female reproductive disorders, when compared to their healthy counterparts [82].

The reduction of plasma and FF level of vitamin E and of beta carotene that accompanies the less successful reproductive outcome, parallels the reduced antioxidant scavenging properties reported in ageing. In the FF of reproductively aged women, the SOD activity increased, catalase and GST decreased and both GPx and GSSG-Reductase exhibited a slight increase [72]; in addition, in older women, there was a drop in the FF concentration of hemopexin, which binds and clears the iron containing heme [83] and some evidence indicates that the response to enhanced local oxidation is sluggish compared to younger women [84]. Not surprisingly, culturing bovine oocytes with FF from older animals, and containing enhanced AGEs level, resulted in accelerated oocyte nuclear maturation, enhanced ROS production and abnormal fertilization [85].

So, in parallel to the plasma, the complex mixture of biomolecules present in the FF is indicative of their involvement in multifaceted biological processes. We are convinced that a part of them modulates the local oxidative balance by way of the properties of their cysteine residues. How the modulation is exerted or how the molecules involved are recruited is essentially unknown but is likely to aim at the provision of appropriate conditions for oocytes to be fertilized. It is interesting that when sensing environmental oxidative challenges, cells activate thiol production mechanisms to buffer the imbalance and regain homeostasis [84].

### Reproductive disorders and perspective of antioxidant intervention

An important part of the discussion about ROS effects on cells relate to their ill-separated dual, harmful or signaling, properties. Also difficult to verify is whether the harmful effects of oxidation antedate well-known disorders or a previously established disorder provides a favorable field for oxidative harmful consequences. This difficulty may underlie the controversies on the effects of oxidation in the reproductive success [86–88].

As oxidation is harmful, it is reasonable to anticipate that the administration of antioxidants will provide protection from excessive oxidation and health improvement. Actually, while the provision of some antioxidants as nutritional supplements to fulfil the recommended dietary allowance (RDA) is beneficial in specific conditions as childhood, pregnancy and perimenopause, their administration in amount above RDA does not support, and sometimes contradicts, what was reasonably expected.

In fact, consistent results indicate that mortality is significantly increased when Beta-carotene, vitamin A and vitamin E are administered in amount above the RDA [89]. In addition, antioxidant supplementation with vitamin C, vitamin E or beta carotene did not offer overall benefits for the primary prevention of cancer incidence or mortality [90] and, instead, they may have a role in the establishment of colorectal cancer [91].

In the area of reproduction, polycystic ovarian syndrome (PCOS) and endometriosis, two major disorders that limit reproductive outcome, have been related to local unbalanced oxidation.

In c.o. cells from patients with PCOS, the ratios of NAD+/NADH and NADP+/NADPH were higher, the ratio of GSH/GSSH was lower and the mitochondrial ROS production, assessed by the fluorescence emission of 2′,7′-Dichlorofluorescein was higher, all suggesting enhanced oxidative stress [92].

Endometriosis is another condition where, apart from some controversy [93], enhanced oxidation was recognized [41, 94–96]. In endometriosis, under the action of the cyclical hormonal changes, an inflammatory reaction is triggered in the proximity of ectopic endometrial cell implants, resulting in attraction of inflammatory cells that activate ROS production by NADPH oxidases. In time, because the inflammatory reaction is cyclical, local structural and functional abnormalities ensue.

In endometriosis, both the elevated ROS production and the reduction of antioxidants were reported. In fact, SOD and vitamin E levels in FF from women with endometriosis were lower, and ROS were higher, compared to controls [97]. In addition, the reduced GSH concentration found in endometriosis FF, compared to controls, was also associated to inferior embryo quality [70].

Moreover, when FF samples from women with endometriosis, who had unsuccessful implantation, were compared with FF samples from women whose implantation was successful, increased concentration of ROS, nitric oxide and malonaldehyde was verified, in contrast to other molecules involved in oxidative stress alleviation [98]. Interestingly, FF albumin and hemopexin, both implicated in oxidative burden mitigation, were identified as distinct proteins in women with endometriosis who conceived, compared to women with endometriosis who did not conceive [99]. These findings indicate that the FF of endometriosis patients has insufficient antioxidant capacity, likely as consequence of extensive FF thiol groups oxidation [70].

Similarly, women whose diagnosis was tubal obstruction or mild tubal disorders and who had successful pregnancies, showed lower concentration of oxidant molecules in the FF when compared with cases of unsuccessful pregnancy [98].

In view of the association of reduced FF antioxidant capacity with diminished reproductive success, and experimental data favoring beneficial effects of antioxidant administration [100], some studies addressed the effects of antioxidant supplementation in humans.

It was reported that the administration of a micronutrient mixture (including vitamins C and E and folic acid,) prior to FF collection, resulted in reduced oxidation of FF components [101] and even increased FF TAC, free thiol residues and the number of viable oocytes [83]. In addition, vitamin C and E intake within the RDA range, compared to intake bellow the RDA, resulted in amelioration of fertilization rate and improvement of early embryo structural fitness [102] and also in a drop of FF myeloperoxidase assessed in women diagnosed with severe endometriosis [103]. Administration of other compounds with antioxidant properties as folate [104] or with D-chiro-inositol phosphoglycan [105] resulted in decrement of FF homocysteine level and in increased free thiol groups of FF proteins, respectively; in both, improvement of oocyte quality was found. A similar change in FF free thiols level was noticed in reproductively aged women upon administration of a micronutrient supplementation containing vitamins C and E and folate [106].

In general, these results favor the existence of a beneficial effect from the use of antioxidant supplementation. However, limitations remain because of the number of subjects enrolled in the studies and the difficulty to verify the precise amount of administered antioxidant.

## Conclusions

Along approximately 3 months, local and systemic signals promote the growth of the ovarian follicle and the establishment of a biomolecule rich fluid compartment.

The structural organization of the COC endows the FF with the ability to supply the oocyte and c.o. cells with a variety of compounds. Among these, the presence and activity of important enzymatic and non-enzymatic antioxidants reflects FF involvement in harmful ROS effects prevention. Less clear is the role of ROS in local biological processes regulation but, the FF volume and rich composition in molecules that modulate redox reactions, that is noticed in pre-ovulatory follicles, suggest that they have a relevant intervention in oocyte growth and maturation.

Current data indicates that an important part of antioxidant buffering effect occurs upon engagement of specific cysteine residues that oxidize rapidly; in this sense, such proteins behave as redox sensors [65], a view that could be extended to albumin in FF because of its abundance and its cysteine mediated antioxidant properties. For other, less concentrated FF proteins, one may speculate that their cysteine residues exert similar roles for self-protection and for local antioxidation. Thus, similarly to the proposed contribution of the intracellular highly concentrated actin [107], cysteines in FF proteins might work as sensors and then as buffers of oxidative stress.

This is an exciting and growing area of research [108, 109]. In the particular field of reproduction, it will provide additional knowledge of a critically regulated set of steps, as is the oocyte maturation process, whose main intervening growth factors are the cysteine rich mature oocyte secretory factors BMP15 and GDF9.

## Abbreviations

AGEs: Advanced glycation end products
BMP15: Bone morphogenetic protein 15
c.o.: *Cumulus oophorus*
CAT: Catalase
cGMP: Cyclic Guanosine Monophosphate cAMC Cyclic Adenosine Monophosphate
COC: Cumulus oocyte complex
CoQ: Coenzyme Q10
FF: Follicular fluid
GDF9: Growth differentiation factor 9
GPx: Glutathione peroxidase
GSH: Reduced Glutathione
GSSG: Oxidized Glutathione
GST: Glutathione S-transferase
LH: Luteinizing Hormone
OSFs: Oocyte secreted factors
PCOS: Polycystic ovarian syndrome
Prx: Peroxiredoxins
ROS: Reactive oxygen species
SOD: Superoxide dismutase
TAC: Total antioxidant capacity
TGFβ: Transforming Growth Factor Beta
UA: Uric acid

## References

[1] Gougeon A (2004). Dynamics of human follicular growth: morphologic, dynamic, and functional aspects. Ovary.

[2] Eppig JJ, Leung PCK, Adashi EY (2004). CHAPTER 7 - Regulation of Mammalian Oocyte Maturation A2.

[3] Zeleznik AJ, Adashi EY, Leung PCK (2004). CHAPTER 3 - Dynamics of Primate Follicular Growth: A Physiological Perspective A2. The Ovary.

[4] Albertini DF, Rider V (1994). Patterns of intercellular connectivity in the mammalian cumulus-oocyte complex. Microsc Res Tech.

[5] Albertini DF (2001). Cellular basis for paracrine regulation of ovarian follicle development. Reproduction.

[6] Li R (2000). Oocyte-secreted factor(s) determine functional differences between bovine mural granulosa cells and cumulus cells. Biol Reprod.

[7] Russell DL (2016). Bidirectional communication between cumulus cells and the oocyte: Old hands and new players?. Theriogenology.

[8] Ozawa M (2010). Cumulus cell-enclosed oocytes acquire a capacity to synthesize GSH by FSH stimulation during in vitro maturation in pigs. J Cell Physiol.

[9] Mori T, Amano T, Shimizu H (2000). Roles of gap junctional communication of cumulus cells in cytoplasmic maturation of porcine oocytes cultured in vitro. Biol Reprod.

[10] Tatemoto H (2004). Protection of porcine oocytes against cell damage caused by oxidative stress during in vitro maturation: role of superoxide dismutase activity in porcine follicular fluid. Biol Reprod.

[11] Tatemoto H, Sakurai N, Muto N (2000). Protection of porcine oocytes against apoptotic cell death caused by oxidative stress during In vitro maturation: role of cumulus cells. Biol Reprod.

[12] Matos L (2009). Superoxide dismutase expression in human cumulus oophorus cells. Mol Hum Reprod.

[13] Eppig JJ, Wigglesworth K, Pendola FL (2002). The mammalian oocyte orchestrates the rate of ovarian follicular development. Proc Natl Acad Sci U S A.

[14] Gilchrist RB (2006). Molecular basis of oocyte-paracrine signalling that promotes granulosa cell proliferation. J Cell Sci.

[15] Juengel JL, McNatty KP (2005). The role of proteins of the transforming growth factor-beta superfamily in the intraovarian regulation of follicular development. Hum Reprod Update.

[16] Laitinen M (1998). A novel growth differentiation factor-9 (GDF-9) related factor is co-expressed with GDF-9 in mouse oocytes during folliculogenesis. Mech Dev.

[17] Peng J (2013). Growth differentiation factor 9:bone morphogenetic protein 15 heterodimers are potent regulators of ovarian functions. Proc Natl Acad Sci U S A.

[18] Mottershead DG (2015). Cumulin, an Oocyte-secreted Heterodimer of the Transforming Growth Factor-beta Family, Is a Potent Activator of Granulosa Cells and Improves Oocyte Quality. J Biol Chem.

[19] Dong J (1996). Growth differentiation factor-9 is required during early ovarian folliculogenesis. Nature.

[20] Elvin JA (1999). Paracrine actions of growth differentiation factor-9 in the mammalian ovary. Mol Endocrinol.

[21] Yan C (2001). Synergistic roles of bone morphogenetic protein 15 and growth differentiation factor 9 in ovarian function. Mol Endocrinol.

[22] Gilchrist RB, Lane M, Thompson JG (2008). Oocyte-secreted factors: regulators of cumulus cell function and oocyte quality. Hum Reprod Update.

[23] Sugiura K (2007). Oocyte-derived BMP15 and FGFs cooperate to promote glycolysis in cumulus cells. Development.

[24] Dragovic RA (2005). Role of oocyte-secreted growth differentiation factor 9 in the regulation of mouse cumulus expansion. Endocrinology.

[25] Su YQ (2008). Oocyte regulation of metabolic cooperativity between mouse cumulus cells and oocytes: BMP15 and GDF9 control cholesterol biosynthesis in cumulus cells. Development.

[26] Sutton-McDowall ML (2015). Redox and anti-oxidant state within cattle oocytes following in vitro maturation with bone morphogenetic protein 15 and follicle stimulating hormone. Mol Reprod Dev.

[27] Diaz FJ, Wigglesworth K, Eppig JJ (2007). Oocytes determine cumulus cell lineage in mouse ovarian follicles. J Cell Sci.

[28] Wigglesworth K (2015). Transcriptomic diversification of developing cumulus and mural granulosa cells in mouse ovarian follicles. Biol Reprod.

[29] Zhang M (2010). Granulosa cell ligand NPPC and its receptor NPR2 maintain meiotic arrest in mouse oocytes. Science.

[30] Lin YC (2003). Human oocyte maturity in vivo determines the outcome of blastocyst development in vitro. J Assist Reprod Genet.

[31] Balaban B, Urman B (2006). Effect of oocyte morphology on embryo development and implantation. Reprod BioMed Online.

[32] Ambekar AS (2013). Proteomic analysis of human follicular fluid: a new perspective towards understanding folliculogenesis. J Proteome.

[33] Kushnir MM (2016). Exploratory study of the association of steroid profiles in stimulated ovarian follicular fluid with outcomes of IVF treatment. J Steroid Biochem Mol Biol.

[34] Angelucci S (2006). Proteome analysis of human follicular fluid. Biochim Biophys Acta.

[35] Twigt J (2012). Proteomic analysis of the microenvironment of developing oocytes. Proteomics.

[36] Hanrieder J (2008). Proteomic analysis of human follicular fluid using an alternative bottom-up approach. J Proteome Res.

[37] Jiang JY (2002). Follicular microvasculature in the porcine ovary. Cell Tissue Res.

[38] Schweigert FJ (2006). Peptide and protein profiles in serum and follicular fluid of women undergoing IVF. Hum Reprod.

[39] Bianchi L (2013). A methodological and functional proteomic approach of human follicular fluid en route for oocyte quality evaluation. J Proteome.

[40] Espey LL (1994). Current status of the hypothesis that mammalian ovulation is comparable to an inflammatory reaction. Biol Reprod.

[41] Agarwal A (2012). The effects of oxidative stress on female reproduction: a review. Reprod Biol Endocrinol.

[42] Duan J, Kasper DL (2011). Oxidative depolymerization of polysaccharides by reactive oxygen/nitrogen species. Glycobiology.

[43] Halliwell B, Gutteridge JMC. Free Radicals in Biology and Medicine. 4th Ed., New York: Oxford University Press Inc; 2007.

[44] Naskalski JW, Bartosz G (2000). Oxidative modifications of protein structures. Adv Clin Chem.

[45] Mary J (2004). Enzymatic reactions involved in the repair of oxidized proteins. Exp Gerontol.

[46] Halliwell B (2007). Biochemistry of oxidative stress. Biochem Soc Trans.

[47] Okado-Matsumoto A, Fridovich I (2001). Subcellular distribution of superoxide dismutases (SOD) in rat liver: Cu, Zn-SOD in mitochondria. J Biol Chem.

[48] Weisiger RA, Fridovich I (1973). Mitochondrial superoxide dismutase. Site of synthesis and intramitochondrial localization. J Biol Chem.

[49] Marklund SL (1982). Human copper-containing superoxide dismutase of high molecular weight. Proc Natl Acad Sci U S A.

[50] Sies H (2014). Role of metabolic H2O2 generation: redox signaling and oxidative stress. J Biol Chem.

[51] Mates JM, Perez-Gomez C, Nunez de Castro I (1999). Antioxidant enzymes and human diseases. Clin Biochem.

[52] Arthur JR (2000). The glutathione peroxidases. Cell Mol Life Sci.

[53] Chaudiere J, Ferrari-Iliou R (1999). Intracellular antioxidants: from chemical to biochemical mechanisms. Food Chem Toxicol.

[54] Turunen M, Olsson J, Dallner G (2004). Metabolism and function of coenzyme Q. Biochim Biophys Acta.

[55] Kand’ar R, Zakova P, Muzakova V (2006). Monitoring of antioxidant properties of uric acid in humans for a consideration measuring of levels of allantoin in plasma by liquid chromatography. Clin Chim Acta.

[56] Wu G (2004). Glutathione metabolism and its implications for health. J Nutr.

[57] Levine M (1986). New concepts in the biology and biochemistry of ascorbic acid. N Engl J Med.

[58] Rumsey SC, Levine M (1998). Absorption, transport, and disposition of ascorbic acid in humans. J Nutr Biochem.

[59] Alej (2013). The Exogenous Antioxidants, in Oxidative Stress and Chronic Degenerative Diseases - A Role for Antioxidants.

[60] Joshi R (2001). Free radical scavenging behavior of folic acid: evidence for possible antioxidant activity. Free Radic Biol Med.

[61] Sies H (2007). Total antioxidant capacity: appraisal of a concept. J Nutr.

[62] Sundaresan M (1995). Requirement for generation of H2O2 for platelet-derived growth factor signal transduction. Science.

[63] Shkolnik K (2011). Reactive oxygen species are indispensable in ovulation. Proc Natl Acad Sci U S A.

[64] Prasad S (2016). Impact of stress on oocyte quality and reproductive outcome. J Biomed Sci.

[65] Biswas S, Chida AS, Rahman I (2006). Redox modifications of protein-thiols: emerging roles in cell signaling. Biochem Pharmacol.

[66] Ghezzi P, Chan P (2017). Redox Proteomics Applied to the Thiol Secretome. Antioxid Redox Signal.

[67] Janssen-Heininger YM (2008). Redox-based regulation of signal transduction: principles, pitfalls, and promises. Free Radic Biol Med.

[68] Boronat S, Domenech A, Hidalgo E (2017). Proteomic Characterization of Reversible Thiol Oxidations in Proteomes and Proteins. Antioxid Redox Signal.

[69] Finkel T (2011). Signal transduction by reactive oxygen species. J Cell Biol.

[70] Choi YS (2015). Alteration in the intrafollicular thiol-redox system in infertile women with endometriosis. Reproduction.

[71] Sabatini L (1999). Superoxide dismutase activity in human follicular fluid after controlled ovarian hyperstimulation in women undergoing in vitro fertilization. Fertil Steril.

[72] Carbone MC (2003). Antioxidant enzymatic defences in human follicular fluid: characterization and age-dependent changes. Mol Hum Reprod.

[73] Kishi I (2016). Thioredoxin, an antioxidant redox protein, in ovarian follicles of women undergoing in vitro fertilization. Endocr J.

[74] Da Broi MG, et al. Increased concentration of 8-hydroxy-2′-deoxyguanosine in follicular fluid of infertile women with endometriosis. Cell Tissue Res. 2016;366(1):231–42. 10.1007/s00441-016-2428-4 27250533

[75] Otsuki J (2012). The influence of the redox state of follicular fluid albumin on the viability of aspirated human oocytes. Syst Biol Reprod Med.

[76] Elizur SE (2014). Reactive oxygen species in follicular fluid may serve as biochemical markers to determine ovarian aging and follicular metabolic age. Gynecol Endocrinol.

[77] Kushnir MM (2012). Protein and steroid profiles in follicular fluid after ovarian hyperstimulation as potential biomarkers of IVF outcome. J Proteome Res.

[78] Pajares M (2015). Redox control of protein degradation. Redox Biol.

[79] Bedaiwy MA (2012). Effect of follicular fluid oxidative stress parameters on intracytoplasmic sperm injection outcome. Gynecol Endocrinol.

[80] Pasqualotto EB (2004). Effect of oxidative stress in follicular fluid on the outcome of assisted reproductive procedures. Fertil Steril.

[81] Das S (2006). Reactive oxygen species level in follicular fluid--embryo quality marker in IVF?. Hum Reprod.

[82] Palini S (2014). Influence of ovarian stimulation for IVF/ICSI on the antioxidant defence system and relationship to outcome. Reprod BioMed Online.

[83] Hashemitabar M (2014). A proteomic analysis of human follicular fluid: comparison between younger and older women with normal FSH levels. Int J Mol Sci.

[84] Watson WH (2016). Differential Regulation of the Extracellular Cysteine/Cystine Redox State (EhCySS) by Lung Fibroblasts from Young and Old Mice. Oxidative Med Cell Longev.

[85] Takeo S, et al. Age-associated deterioration in follicular fluid induces a decline in bovine oocyte quality. Reprod Fertil Dev. 2016. doi:10.1071/RD15228. 10.1071/RD15228 26829061

[86] Jozwik M (1999). Oxidative stress markers in preovulatory follicular fluid in humans. Mol Hum Reprod.

[87] Attaran M (2000). The effect of follicular fluid reactive oxygen species on the outcome of in vitro fertilization. Int J Fertil Womens Med.

[88] Oral O (2006). The effects of oxidative stress on outcomes of assisted reproductive techniques. J Assist Reprod Genet.

[89] Bjelakovic G, Nikolova D, Gluud C (2013). Meta-regression analyses, meta-analyses, and trial sequential analyses of the effects of supplementation with beta-carotene, vitamin A, and vitamin E singly or in different combinations on all-cause mortality: do we have evidence for lack of harm?. PLoS One.

[90] Lin J (2009). Vitamins C and E and beta carotene supplementation and cancer risk: a randomized controlled trial. J Natl Cancer Inst.

[91] Choi JH (2014). Contemporary issues surrounding folic Acid fortification initiatives. Prev Nutr Food Sci.

[92] Zhao H (2015). Metabolism alteration in follicular niche: The nexus among intermediary metabolism, mitochondrial function, and classic polycystic ovary syndrome. Free Radic Biol Med.

[93] Gupta S (2008). Pathogenic mechanisms in endometriosis-associated infertility. Fertil Steril.

[94] Da Broi MG, Navarro PA (2016). Oxidative stress and oocyte quality: ethiopathogenic mechanisms of minimal/mild endometriosis-related infertility. Cell Tissue Res.

[95] Carvalho LF (2012). Oxidative stress biomarkers in patients with endometriosis: systematic review. Arch Gynecol Obstet.

[96] Regiani T (2015). Follicular fluid alterations in endometriosis: label-free proteomics by MS(E) as a functional tool for endometriosis. Syst Biol Reprod Med.

[97] Liu F (2013). The expression and role of oxidative stress markers in the serum and follicular fluid of patients with endometriosis. Clin Exp Obstet Gynecol.

[98] Singh AK (2013). Markers of oxidative stress in follicular fluid of women with endometriosis and tubal infertility undergoing IVF. Reprod Toxicol.

[99] Lo Turco EG (2013). Proteomic analysis of follicular fluid from women with and without endometriosis: new therapeutic targets and biomarkers. Mol Reprod Dev.

[100] Giorgi VS (2016). N-Acetyl-Cysteine and l-Carnitine Prevent Meiotic Oocyte Damage Induced by Follicular Fluid From Infertile Women With Mild Endometriosis. Reprod Sci.

[101] Ozkaya MO, Naziroglu M (2010). Multivitamin and mineral supplementation modulates oxidative stress and antioxidant vitamin levels in serum and follicular fluid of women undergoing in vitro fertilization. Fertil Steril.

[102] Kazemi A, Ramezanzadeh F, Nasr-Esfahani MH (2015). The relations between dietary antioxidant vitamins intake and oxidative stress in follicular fluid and ART outcomes. Iran J Reprod Med.

[103] Santanam N, Zoneraich N, Parthasarathy S (2017). Myeloperoxidase as a Potential Target in Women With Endometriosis Undergoing IVF. Reprod Sci.

[104] Szymanski W, Kazdepka-Zieminska A (2003). Effect of homocysteine concentration in follicular fluid on a degree of oocyte maturity. Ginekol Pol.

[105] Piomboni P (2014). Protein modification as oxidative stress marker in follicular fluid from women with polycystic ovary syndrome: the effect of inositol and metformin. J Assist Reprod Genet.

[106] Luddi A (2016). Antioxidants reduce oxidative stress in follicular fluid of aged women undergoing IVF. Reprod Biol Endocrinol.

[107] Castro JP (2013). Actin carbonylation: from cell dysfunction to organism disorder. J Proteome.

[108] Weerapana E (2010). Quantitative reactivity profiling predicts functional cysteines in proteomes. Nature.

[109] Paulsen CE, Carroll KS (2010). Orchestrating redox signaling networks through regulatory cysteine switches. ACS Chem Biol.

